# Closing the Gap - Integrated Time-Resolved Crystallography at the SwissFEL and Swiss Light Source

**DOI:** 10.1063/4.0000882

**Published:** 2025-10-27

**Authors:** Florian Dworkowski

**Affiliations:** Paul Scherrer Institut, Villigen PSI, Aargau, Switzerland

## Abstract

The SwissFEL currently provides the hard X-ray endstations Alvra, Bernina, and Cristallina. Especially Alvra is a forerunner in the crystallographic community, having successfully performed many serial femtosecond crystallography experiments, using both, high-viscosity extruders (HVE) and GDVN jets as the sample delivery method [1-3]. With the recently commissioned SwissMX experiment based at Cristallina, there is now also a dedicated setup for fixed-target experiment, both in high-throughput and pump-probe mode, with a mixing setup on the horizon. However, available beamtime at FELs is sparse and entry barrier for new teams is high.

To alleviate these issues and close the probe-time-gap in the millisecond-to-second regime, we build the VESPA endstation at the Swiss Light Source (SLS), dedicated to multi-time-resolved serial millisecond crystallography [4], acoustic levitation goniometry [5], and kilohertz data acquisition serial crystallography [6]. The latter allowed us to push the achievable time resolution at a synchrotron source to microseconds, without the need for choppers. In combination with different pump methods, including dedicated *cw* and nanosecond Lasers, as well as temperature control, and ligand mixing, this will enable our research community to investigate an even larger array of protein samples.

We will introduce the recently formed PSI focus team for time resolved crystallography, which is dedicated to facilitating easy access to facilities and instruments, as well as providing training and support for research teams interested to get into the field. We will also present an overview of available techniques and expertise, including results from our experimental portfolio, at both SwissFEL and the SLS, and will present an outlook on novel techniques and instruments, especially in light of the upcoming SLS 2.0 upgrade.
